# Structuring functional groups of aquatic insects along the resistance/resilience axis when facing water flow changes

**DOI:** 10.1002/ece3.8749

**Published:** 2022-03-26

**Authors:** Bruno S. Godoy, Francisco Valente‐Neto, Luciano L. Queiroz, Luis F. R. Holanda, Fabio O. Roque, Sara Lodi, Leandro G. Oliveira

**Affiliations:** ^1^ 37871 Centre of Aquatic Ecology and Fishery Federal University of Pará Belém Pará Brazil; ^2^ Federal University of Mato Grosso do Sul Campo Grande Brazil; ^3^ Institute of Biomedical Science USP São Paulo Brazil; ^4^ Program of Ecology and Evolution UFG Goiânia Brazil; ^5^ Centre for Tropical Environmental and Sustainability Science (TESS) James Cook University Cairns Queensland Australia; ^6^ Biological Science Institute UFG Goiânia Brazil

**Keywords:** Bayesian inference, Cerrado streams, functional trait, hierarchical model, resistance–resilience axis, space‐state design

## Abstract

Understanding how differences in intensity and frequency of hydrological disturbances affect the resistance and resilience of aquatic organisms is key to manage aquatic systems in a fast‐changing world. Some aquatic insects have strategies that improve the permanence (resistance), while others use strategies that favor recolonization (resilience). Therefore, we carried out a manipulative experiment to understand the influence of functional characteristics of aquatic insects in their permanence and recolonization against hydrological disturbances in streams in the biodiversity hotspot of the Cerrado of Brazil. We placed 200 artificial substrates in five streams and submitted them to changing water flow regimes that differed both in frequency and intensity, and we observed the response of the aquatic community for 39 days. We used a hierarchical Bayesian approach to estimate the probabilities of permanence and recolonization of each life strategy group (nine groups). We observed that the most intense changes in the water flow tended to affect the permanence of almost all groups, but the intensity of this effect reduced over time. On the other hand, less frequent disturbances, regardless of intensity, tended to reduce the permanence of most groups of aquatic insects over time. The different effects of disturbance intensity may have been related to a greater recolonization capacity of some groups. The results we present are worrisome in a scenario of reduced riparian vegetation around streams and with the expectation of precipitation becoming more concentrated in shorter periods of time due to climate change in the Cerrado hotspot, reducing the occurrence of many groups of aquatic insects in their habitat, particularly those with traits associated with resistance against hydrological disturbance.

## Graphical abstract

Effects of experimental disturbance in intensity and frequency of water flow on the probability of permanence of aquatic insects in Cerrado streams.

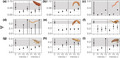

## 1 INTRODUCTION

The high rates of change in natural environments direct and indirectly caused by human activities may exert negative effects on terrestrial and aquatic ecosystems (Secretariat of the Convention on Biological Diversity, 2020). In lotic environments, human modifications at local (e.g., removal of riparian vegetation), regional (e.g., river damming), and global scales (e.g., climate change) can change water flow, and regularity of flood and flash flood events (Allan, 2004; Arias et al., 2021; Dudgeon et al., 2006; Poff et al., 2007). Changes made to the surroundings of lotic systems, such as the removal of riparian vegetation and the linearization of water courses, reduce the area available for water infiltration, and increase the occurrence of flood and flash flood events (Resh et al., 1988). In tropical regions, precipitation tends to become more concentrated in shorter periods of time, and, consequently, the amounts of heavy precipitation events are expected to increase in the coming years (Arias et al., 2021; Fischer & Knutti, 2015). Then, the flow regime of lotic systems will change due to an expected increase in disturbance events caused by altered precipitation. Manipulative experiments are key for understanding the mechanisms behind the responses of biodiversity to environmental changes because they can separate the effects of different drivers related to the disturbance.

For a long time, aquatic environments have been undergoing hydrological disturbances, and studies addressing this topic are essential to understand ecosystem dynamics and biodiversity patterns (Lake, 2000; Melo et al., 2003; Shabarova et al., 2021; Sutherland & Freckleton, 2013; Townsend, Scarsbrook, et al., 1997). Historically, knowledge on the effects of hydrological disturbances on biodiversity (e.g., water withdrawal or flash floods) usually focus on taxonomic measures such as species richness and beta diversity indices (Death, 1996; Lake, 2000; Melo & Froehlich, 2004). However, environmental disturbances can affect species differently depending on their functional traits, which still needs to be further studied for aquatic systems (Menezes et al., 2010). Community functionality is an important component of biodiversity, once function considers not only the identity of organisms but also the physiological, behavioral, and/or ecological responses of species to the surrounding environment (Verberk et al., 2013).

Traits related to permanence and recolonization are important for aquatic communities when facing hydrological disturbances. Permanence and recolonization traits can be inserted in resistance/resilience axis, relative to the organism occurrence (Sarremejane et al., 2020; Townsend, Dolédec, et al., 1997; Verberk et al., 2013; Wilkes et al., 2020). In general terms, the trait sets related to resilience are different from that related to resistance. Resistance traits, such as morphological adaptations for fixation on substrates and body shape, allow organisms to maintain persistent population sizes when resisting disturbances (Mazzucco et al., 2015). Resilience traits are related to life cycle length such that short life cycles enable faster recolonization in newly impacted environments (Ribera et al., 2001). The relationship between resistance and resilience traits is a trade‐off; organisms that have a high resistance to disturbance tend to present reduced resilience characteristics, whereas organisms with low resistance to disturbance tend to have increased resilience (Ferzoco, 2019; Kappes et al., 2014).

Functional traits not directly related to resistance/resilience (e.g., food acquisition) can shape communities (Wilkes et al., 2020). The role of these other traits on community structure may depend on hydrological disturbances, being more important in low‐disturbance conditions (Walters, 2011). On the other hand, resistance–resilience trade‐offs become evident with greater disturbance, either in the form of intensity and/or frequency. An increased environmental disturbance tends to reduce the importance of traits related to competitive interactions shaping communities in a given environment (Lopez et al., 2016). If environmental disturbance is high, then aquatic communities will tend to be structured according to traits related to resistance and resilience. In such circumstances, we can expect organisms with different traits related to resistance and resilience to have different responses to hydrological disturbances (Kappes et al., 2014). Intensity and frequency of disturbance determines if resistance or resilience traits are more advantageous.

Streams in the Cerrado biome have key elements for manipulative experiments, mainly related to the effect that changes in water flow can have on aquatic organisms. The Cerrado is among the most threatened tropical biomes in the world and it is among the top five global biodiversity hotspots (Mittermeier et al., 2005; Myers et al., 2000). Headwater streams in the Cerrado are generally recognized as being sensitive to anthropogenic alterations, particularly due to land use changes. This biome is crucial to maintain the dynamics of the large water resources in South America, such as the Prata and Amazonas Rivers. In addition, 70% of the Brazil's energy production comes from hydroelectric power generation in rivers located within the Cerrado domain (Oliveira et al., 2014). Streams in the Cerrado experience extremely short hydrological pulses due to storms, locally known as “trombas d’água” (term literally translated to “waterspout”). Moreover, extreme weather, extreme hydrological events, wildfires, and more severe dry seasons are expected to become more frequent in the region (Arias et al., 2021). These general issues regarding the importance of highly diverse headwater streams require monitoring networks that are able to indicate if there is enough information to detect any trends of declining biodiversity.

Under this perspective, we conducted a manipulative hydrological experiment in streams of the biodiversity hotspot of the Cerrado of Brazil to test whether modifying stream flow changes the rate of occurrence and recolonization of aquatic insects with different functional traits. We choose to use aquatic insects because there are relevant studies about the dependence of this organism to environment conditions with their functional traits and abundance (Brasil et al., 2020; Godoy, Simião‐Ferreira, et al., 2016). We tested two general hypotheses: (i) that an increased disturbance intensity would reduce the persistence of organisms in the substrates, and (ii) that higher disturbance frequency would negatively affect organisms that maximize the permanence in the environments. To test these two hypotheses, we categorized the aquatic insects into nine groups with similar functional traits, both morphological and ecological. For each group, we established hypotheses related to the permanence and recolonization parameters (see Table 2, in data analysis, for details and references that support our expectations). We designed an experiment in a space‐state design (Pedersen et al., 2011), changing both intensity and frequency of water flow, and evaluated the occurrence of the genera. We also estimated how these changes influenced the probabilities of permanence and recolonization of each functional group using a statistical model with Bayesian inference.

## 2 METHODS

### 2.1 Space‐state experiment

We used an experimental design based on the space‐state concept. A space‐state design follows the dynamics of a variable of interest during a fractioned period of time (Pedersen et al., 2011). A relevant point for this method is that the current condition of the variable is bound to its past condition. Changes in the variable after the beginning of the experiment may be addressed to assess the experimental modifications. The parameters permanence and recolonization of the genera were our variables of interest in this study. The permanence parameter was related to the resistance of the genera to the disturbance, and for the other hand, the recolonization parameter can be used to infer the resilience.

We used artificial substrates (AS) with standardized area, weight, and volume. Each substrate was composed of washed river pebbles and eight leaves of *Tapirira guianensis* Aubl. (Anacardiaceae), a common plant in the region, with a standardized leaf area of 25.63 cm^2^, all packed within a 500 cm^3^ (10 cm × 10 cm × 5 cm) rectangular wire mold. AS are efficient for experiments of aquatic insect colonization in Cerrado streams, constituting a well delimited sample unit (Carvalho & Uieda, 2006; Godoy, Queiroz, et al., 2016). We used a 0.225 mm hand sieve when sampling the AS and fixed the samples in 80% ethanol. The organisms were identified to the highest possible taxonomic resolution (genus or family level) (Inês et al., 2007; Manzo, 2005; Manzo & Archangelsky, 2008; Merritt et al., 2008; Oliveira, 2006; Pes et al., 2005; Trivinho‐Strixino & Strixino, 1995; Wiggins, 1977).

### 2.2 Study area

The experiment was conducted in five streams at the Pirancajuba River basin, within and near the Silvânia National Forest (FLONA Silvânia, 48º38’50” W, 16º38’11” S). FLONA Silvânia is an environmental conservation unit, located in the eastern region of the state of Goiás, with 486.37 ha that preserves most of the natural characteristics of the Cerrado (ICMBio, 2015). The region has a warm and semi‐humid tropical climate, strongly characterized by a rainy season in the summer (November to March), and a dry winter season with an average duration of 4 months (May to September) (Nimer, 1972). The experiment was conducted during the dry season of 2010 (from July to September), season which maximizes sampling of aquatic insects in Cerrado streams (Bispo & Oliveira, 2007; Bispo et al., 2004).

The five streams have similar water velocity, average depth, streambed composition, and a prevalence of sand, silt, and small‐sized stones. Water flow was the hydrologic measure with the greatest variation (coefficient of variation: 108%), followed by channel width (CV: 67%). Average depth and water velocity presented more constant values (Table 1).

**TABLE 1:** Physical variables measured in the streams where the experiment was conducted

Stream	Channel width (m)	Average depth (m)	Water velocity (m s^−1^)	Hydrological flow (m^3^ s^−1^)
1	2.95	0.14	0.39	0.16
2	3.40	0.17	0.31	0.18
3	0.80	0.10	0.24	0.02
4	1.15	0.08	0.20	0.02
5	0.90	0.06	0.18	0.01

### 2.3 Sampling design

We began to sample the experimental units 23 days from the beginning of the experiment. We removed one control and three treatments AS (F1, F2, and F3) from each stream, also using a hand sieve, and collected a total of four experimental units per stream per sampling activity. Other four AS were again removed 4 days later until the 39th day of the experiment, when the fifth and last AS were removed from each stream. At this stage of the experiment, the AS were always collected before applying the disturbance. A total of 200 experimental units were collected after the frequency and intensity experiments (20 units per stream, 5 streams, and 2 experimental batteries).

### 2.4 Immediate effect

We added two substrates to each stream to test the immediate effect of the disturbance on the communities. After 21 days, we collected an experimental unit and subjected another to a disturbance. The 21‐day interval was previously reported as enough for the establishment of a stable aquatic insect community (Godoy, Queiroz, et al., 2016; Landeiro et al., 2010). The disturbance was a subtle increase in water flow using a water pump from a 60 cm distance upstream. The increased water flow for this experimental unit was 60 m^3^ h^−1^ for 10 min. To test the immediate effect, we collected the AS right after the disturbance.

### 2.5 Frequency and intensity effects

**FIGURE 1:**
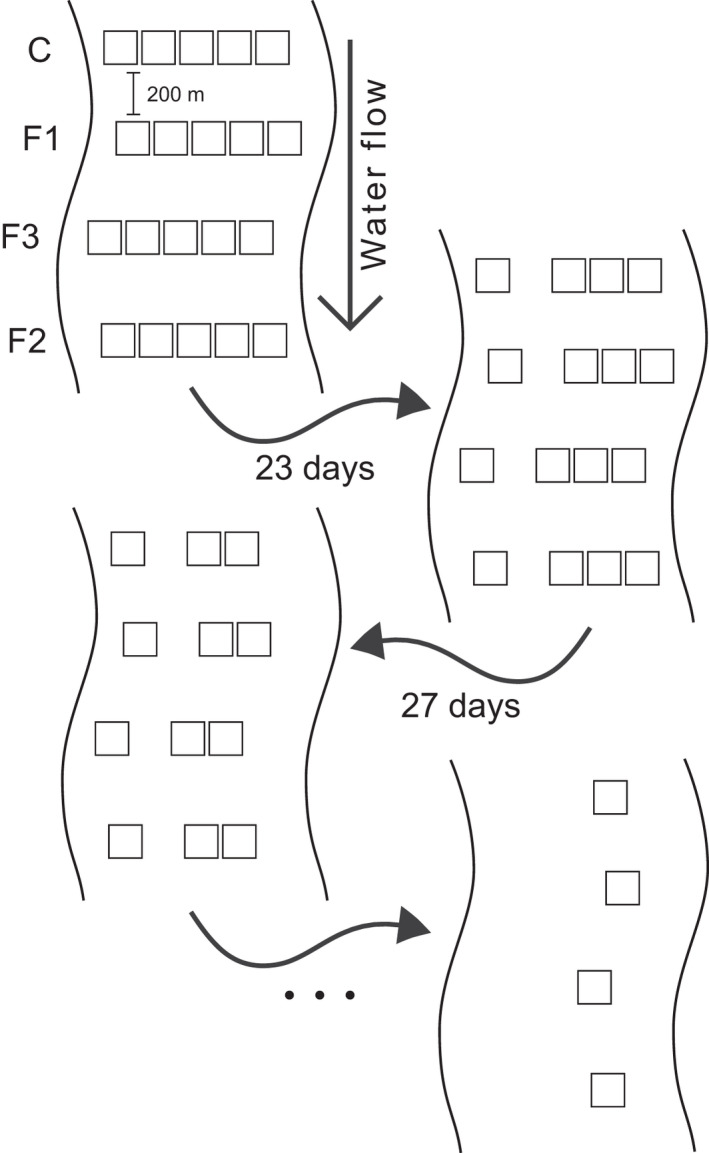
Experimental design of the artificial substrates placed within the streams. A total of five artificial substrates (AS) were placed in four rows transversal to the stream. The frequency of the disturbance (control, F1, F2, or F3) was assigned randomly to each row of each stream. An entire column was randomly selected to be sampled at each sampling procedure. The same design was used for the two times the experiment was conducted, with different intensities of disturbance

A total of 20 AS arranged in 4 rows and 5 columns were introduced to each stream. The lines were allocated transversally to the stream, and the distance between each line was at least 200 m. The distance between columns was irrelevant because, in space‐state design, the spatial autocorrelation between samples of the same time series is desired once the model focuses on changes overtime rather than differences in each sample. We randomly distributed the frequency of disturbance to each line (Figure 1). The frequency of the disturbance (applied in each line separately) was composed of the control, disturbance at every 6 days (F1), disturbance at 3 days (F2), and random disturbance (F3; i.e., 1 control and 3 treatments per stream).

To test the different intensities of disturbance, we conducted the experiment twice. The first time with an increased water flow intensity of 30 m^3^ h^−1^ for 10 min on all AS except the control level. The second time the intensity was 60 m^3^ h^−1^ for 10 min. The first battery of the experiment occurred in July and the second in August. The distance among the AS remained the same.

### 2.6 Functional traits

We divided the genera of aquatic insects into nine groups of organisms with different life history strategies. These groups were formed by combinations of morphological (body shape and morphological structures for fixation in the substrate) and reproductive (life cycle length and potential number of reproductive cycles) functional traits relevant for their permanence in and recolonization of streams subject to environmental disturbances (Poff et al., 2006; Sarremejane et al., 2020; Verberk et al., 2013). We used information about the organism's response to environment of other regions (tropical or not) to define these history strategies, since there are relative few studies about this topic in Cerrado streams. The body shape of the insects collected during the experiment was divided into depressed and cylindrical bodies. The morphological structures for fixation were divided into present and absent. The life cycle length was separated into organisms with relatively short and long cycles, and the potential number of reproductive cycles was divided into low and high. The classification into long, short, low, or high for functional traits is relative to the set of aquatic genera species observed in the study, considering the duration of the study (39 days). Thus, when referring to a long life cycle, the implication is that an organism of the group has the potential to survive in the watercourse for the total study period. The same reasoning can be used for the number of potential reproductive cycles.

### 2.7 Data analysis

The data were analyzed using self‐regressive linear models with two parameters of interest, namely the permanence probability of the taxon in the occurrence of the disturbance $\left(\psi \right)$, and the recolonization probability in a substrate without previous occurrence of the taxon $\left(\theta \right)$. The standard model used has the following structure:

$$\begin{array}{cc} O_{t} & ∼\text{Bern}\left(P_{t}\right)\\ P_{t} & =\theta +O_{t‐1}\cdot \psi , \end{array}$$

where *O_t_* was the occurrence of the taxon at time *t*. This model was modified to fit each stage of the experiment. We used Bayesian inference to adjust the models to the data and to estimate the credibility intervals (CI; 95%) for the parameters of interest. All analyses were conducted in R (R Development Core Team, 2020), using the *runjags* package (Denwood, 2016). We calculated the CI using the methodology of resampling by relevance, in a Monte Carlo Markov chain iteration (Andrade & Kinas, 2008; Gelman et al., 2000). In all analyses, we used 5 parallel chains, burning the first 1000 iterations and sampling every 50 iterations, in a total of 5000 chain steps. The priori distributions of all analysis were not informative.

The combinations of the traits of the insects characterize the life history strategies for the organisms (Poff et al., 2006; Sarremejane et al., 2020; Verberk et al., 2013). We elaborated individual hypotheses for each life history strategy because many groups of insects had similar traits. However, the list of trait combinations was not an exhaustive possibility of all combinations, but it was the specific combination for the groups observed in the experiment. The hypotheses are related to estimates of the permanence and recolonization parameters and are based on information on how the functional traits may be affecting these parameters (Table 2).

**TABLE 2:** Classification of the genera of aquatic insects according to groups of functional traits and life history strategy when subjected to disturbance events

Group	Functional traits	Life history strategy	Hypothesis	Organisms
1	Depressed body; Relatively long‐life cycle; Low potential number of reproductive cycles; No developed fixation structure.	Body shape acts as a resistance factor against the disturbance.	ψ high θ low	Coleoptera larvae with depressed body
2	Cylindrical body; Relatively long‐life cycle; Low potential number of reproductive cycles; No developed fixation structure.	Body shape does not confer resistance to the disturbance.	ψ low θ low	Coleoptera larvae with cylindrical body
3	Cylindrical body; Relatively short life cycle; High potential number of reproductive cycles; No developed fixation structure.	Life cycle as a resilience factor.	ψ low θ high	Chironomidae larvae
4	Cylindrical body; Relatively short life cycle; Moderate potential number of reproductive cycles; No developed fixation structure.	Life cycle as a resilience factor.	ψ low θ moderate	Ceratopogonidae larvae
5	Cylindrical body; Relatively short life cycle; High potential number of reproductive cycles; Adhesive buttons for fixation.	Structures of fixation as a resistance factor and life cycle as a resilience factor.	ψ moderate θ high	Empididae and Simuliidae larvae
6	Cylindrical body; Relatively moderate life cycle; Moderate potential number of reproductive cycles; No developed fixation structures.	Organisms sensitive to disturbances by carrying and by injury.	ψ low θ moderate	Baetidae larvae
7	Depressed body; Relatively moderate life cycle; Moderate potential number of reproductive cycles; No developed fixation structures.	Organisms sensitive to disturbances by carrying and by injury, but with hydrodynamic body as a resistance factor.	ψ moderate θ moderate	Leptohyphidae, Leptophlebiidae, and Plecoptera
8	Cylindrical body; Relatively high life cycle; Low potential number of reproductive cycles; Well‐developed hooks for fixation.	Organisms with appendixes (anal claws) as resistance factor.	ψ very high θ low	Gyrinidae (larvae), Megaloptera, and some Trichoptera
9	Cylindrical body; Relatively moderate life cycle; Low potential number of reproductive cycles; Construction of shelters.	Organisms that build shelters to resist the disturbance.	ψ very high θ moderate	Calamoceratidae, Hydroptilidae, Leptoceridae, and Odontoceridae.

#### 2.7.1 Probability of permanence and recolonization

Using the control AS, we estimated the basal values of the parameters for the permanence and recolonization probability for each genus of aquatic insect. The model used in this step was the following modification of the standard model:

(1) $$\begin{array}{cc} O_{i,t} & ∼\text{Bern}\left(P_{i,t}\right)\\ P_{i,t} & =\theta_{g}+O_{i,t‐1}\cdot \psi_{g}\\ \theta_{g} & ∼\text{Beta}\left(\alpha_{1,g},\beta_{1,g}\right)\\ \psi_{g} & ∼\text{Beta}\left(\alpha_{2,g},\beta_{2,g}\right)\\ \alpha_{1,g} & ∼\text{Gamma}\left(0.001,0.001\right)\\ \beta_{1,g} & ∼\text{Gamma}\left(0.001,0.001\right)\\ \alpha_{2,g} & ∼\text{Gamma}\left(0.001,0.001\right)\\ \beta_{1,g} & ∼\text{Gamma}\left(0.001,0.001\right), \end{array}$$

where *O_i_*_,_ *_t_* was the occurrence of genus *i* at time *t*, and *P_i_*_,_ *_t_* was the probability of the occurrence of genus *i* at time *t* given its occurrence at time *t*–1. The parameters α and β in model 2 were the priori distribution of the probabilities of permanence (ψ) and recolonization (θ) of group *g* to which genus *i* belongs.

#### 2.7.2 Immediate effect

To test the immediate effect of the disturbance on aquatic insect communities, we estimated the difference between the probabilities of occurrence of each genus before (*B*) and after (*A*) the water flow change. For this analysis, we used the artificial substrates sampled on day 21. The model used was as follows:

(2) $$\begin{array}{cc} a_{g} & =\frac{b_{g}}{e_{g}}\\ B_{i} & ∼\text{Bin}\left(b_{g},n\right)\\ A_{i} & ∼\text{Bin}\left(a_{g},n\right)\\ b_{g} & ∼\text{Beta}\left(1,1\right)\\ a_{g} & ∼\text{Beta}\left(1,1\right)\\ e_{g} & ∼\text{Gamma}\left(0,0\right)\\ {\text{Effect}}_{g} & =\exp\left(e_{g}\right) \end{array}$$

In model 1, *B_i_* and *A_i_* are the number of occurrences of genera *i* in the streams before and after the disturbance, *n* was the number of possible events for the occurrence (total observations), and Effect*_g_* was the estimated value for the effect of the disturbance on group *g*.

#### 2.7.3 Effects of disturbance on the probability of permanence and recolonization

After the parameters of permanence and basal recolonization of the functional groups were estimated, we used the results of the artificial substrates under different disturbances to assess the effect of water flow changes on genus occurrence. We added a new parameter to the model, which modifies the probability of genera permanence during the experiment. The model used was as follows:

(3) $$\begin{array}{cc} O_{i,k,t} & ∼\text{Bern}\left(P_{i,k,t}\right)\\ P_{i,k,t} & =\theta_{g}+O_{i,k,t‐1}\cdot \psi_{g}\cdot \delta_{k,g}\\ \delta_{k,g} & ∼N\left(0,0.001\right), \end{array}$$

where *O_i_*_,_ *_k_*_,_ *_t_* is the occurrence of genus *i* in treatment *k* at time *t*. In model 3, $\delta_{k,g}$ was the deviation of the probability of permanence of group *g* from treatment *k*, compared to the baseline permanence of the group observed in the control ($\psi_{g}$). We assumed the six factorial interactions by treatment (I_1_.F_1_, I_1_.F_2_, I_1_.F_3_, I_2_.F_1_, I_2_.F_2_, and I_2_.F_3_).

We highlight that the parameters of interest in the study were the hyperparameters estimated in model for ψ and θ. These hyperparameters are calculated using the values for α and β. Thus, each genus has its estimated probability value. However, all are linked to the baseline values of the functional groups in which they were categorized.

## 3 RESULTS

We collected 15,573 individuals of aquatic insects distributed among 92 taxonomic groups. We identified 84 genera in the orders Coleoptera, Diptera, Ephemeroptera, Plecoptera, Trichoptera, and Megaloptera. Eight groups were identified to the family level (Dryopidae, Dysticidae, Gyrinidae, Hydrophilidae, Ptilodactylidae, Ceratopogonidae, Empididae, and Simuliidae). The Chironomidae family was the most well represented, with 38 genera, followed by the order Ephemeroptera (20 genera), Trichoptera (13 genera), and Elmidae (10 genera). The order Plecoptera had only the genus *Anacroneuria* (Perlidae) and we identified the genera *Corydalus* and *Protosialis* in the order Megaloptera.

### 3.1 Immediate effect on the occurrence of the functional groups

**FIGURE 2:**
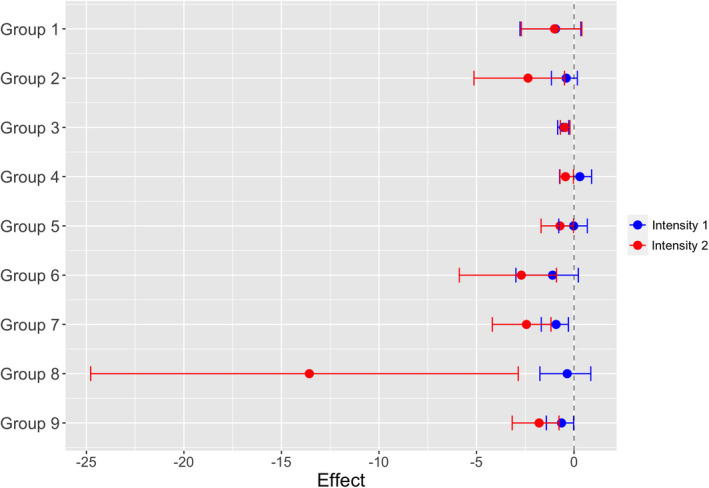
Immediate effect of disturbance on the permanence probability of the functional groups. Effect is the parameter estimated in the model that affects the probability of occurrence

The two intensities of disturbance had different effects on the occurrence of the functional groups (Figure 2). The probability of occurrence reduced for all groups after the strongest disturbance (Intensity 2), except for organisms with depressed body (group 1). The group of organisms with appendixes to fix in the substrate showed the highest reduction in the probability of occurrence at the highest intensity of the disturbance (group 8). For the weakest disturbance (Intensity 1), only Chironomidae, Baetidae, and Trichoptera shelter builders (groups 3, 7, and 9) had a reduced probability of occurrence.

### 3.2 Probability of permanence and basal recolonization

**FIGURE 3:**
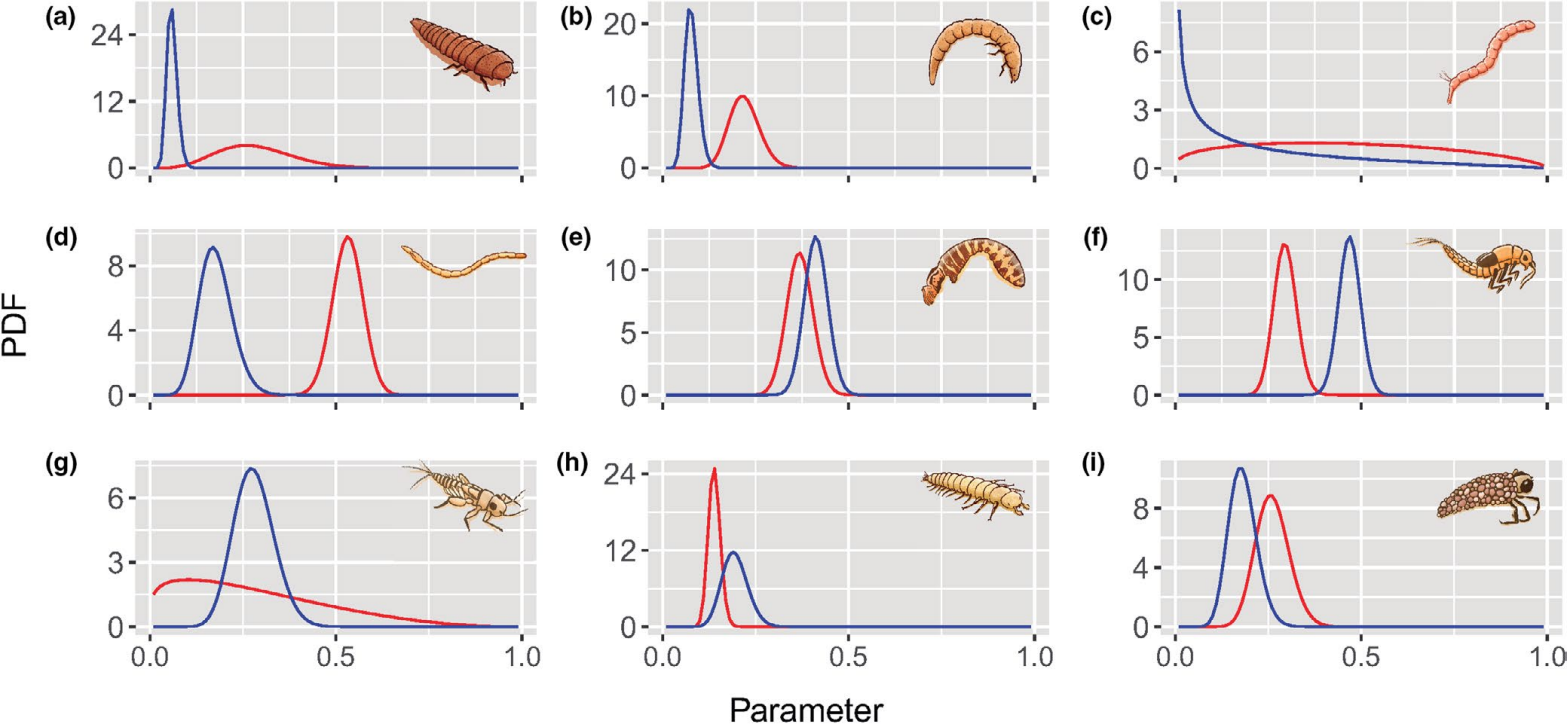
Probability density function (PDF) of permanence (ψ‐blue) and colonization Parameters (θ‐red) for functional groups of aquatic insects, during 39 days in streams of the Brazilian Cerrado. The letters A–I correspond to the functional 1–9 groups (a = Depressed body Coleoptera; b = Cylindrical Coleoptera; c = Chironomidae; d = Ceratopogonidae; e = Empididae and Simuliidae; f = Baetidae; g = Depressed body insects; h = Larvae with anal claws; and i = Trichoptera shelter builders)

Each functional group showed different permanence probability and basal recolonization values (Figure 3). Only the groups that included Chironomidae and Ceratopogonidae larvae (groups 3 and 4) had ψ and θ estimates supported by hypothesized values. The groups including Empididae and Simullidae larvae (group 5), Baetidade (group 6), Leptohyphidae, Leptophlebiidae and Plecoptera (group 7), larvae with anal appendix (group 8) and shelter‐building larvae (group 9) had their hypotheses partially corroborated, and the value of at least one of the parameters was similar to the hypothesized values.

Coleoptera larvae with depressed body presented a moderate probability of recolonization, and a low probability of permanence (Figure 3a). The genera *Macrelmis* had the highest estimated probability of recolonization (Figure A1, in the Appendices). The probability of recolonization for Coleoptera larvae with cylindrical body was estimated between 0.15 and 0.29 (Figure 3b). The genera *Heterelmis*, *Neoelmis*, and the family Ptilodactylidae had the highest probability of recolonization and permanence among the coleoptera (Figure A2).

The group of Chironomidae larvae had the most variable probability of recolonization, with values ranging from 0.12 to 0.86 (Figure 3c). The genera *Rheotanytarsus* presented the highest probabilities of both recolonization and permanence. On the other hand, the genera *Polypedilum* presented a low probability of recolonization, and a high permanence. The genera *Cryptochironomus*, *Stempellinela*, *Stenochironomus*, and *Zavrelliela* presented the opposite pattern, with high probability of recolonization and low permanence (Figure A3). Ceratopogonidae larvae presented a high recolonization rate and low permanence rate, typical of R‐strategist groups (Figure 3d and Figure A4). Empididae and Simuliidae larvae showed lower recolonization rates compared to Ceratopogonidae, but a higher permanence probability (Figure 3e and Figure A5).

In the absence of disturbance, the recolonization probability of fusiform Ephemeroptera nymphs ranged from 0.26 to 0.38 and permanence probabilities were greater than 0.4 (Figure 3f and Figure A6). The insects with depressed bodies showed a highly variable recolonization rate (Figure 3g). *Tricorythopsis* and *Simothraulopsis* showed the lowest recolonization probabilities, while *Leptohyphes*, *Tricorythodes*, and *Anacroneuria* showed high recolonization probabilities, ranging from 0.45 to 0.54 (Figure A7). The probability of recolonization was low for larvae with anal claws (Figure 3h), and the probability of permanence for this group ranged between 0.18 and 0.28, with the lowest value for *Macronema*, and the highest for *Corydalus* (Figure A8). The recolonization probabilities of shelter‐building Trichoptera ranged from 0.19 to 0.36 (Figure 3i), with the lowest value for *Oecetis* and the highest for *Neotrichia*. The probabilities of permanence for this group (shelter‐building Trichoptera) were lower than 0.20, except for the genera *Neotrichia* and *Oecetis*, with probabilities of 0.28 and 0.31, respectively (Figure A9).

### 3.3 Long‐term effects on the probability of permanence for the functional groups

**FIGURE 4:**
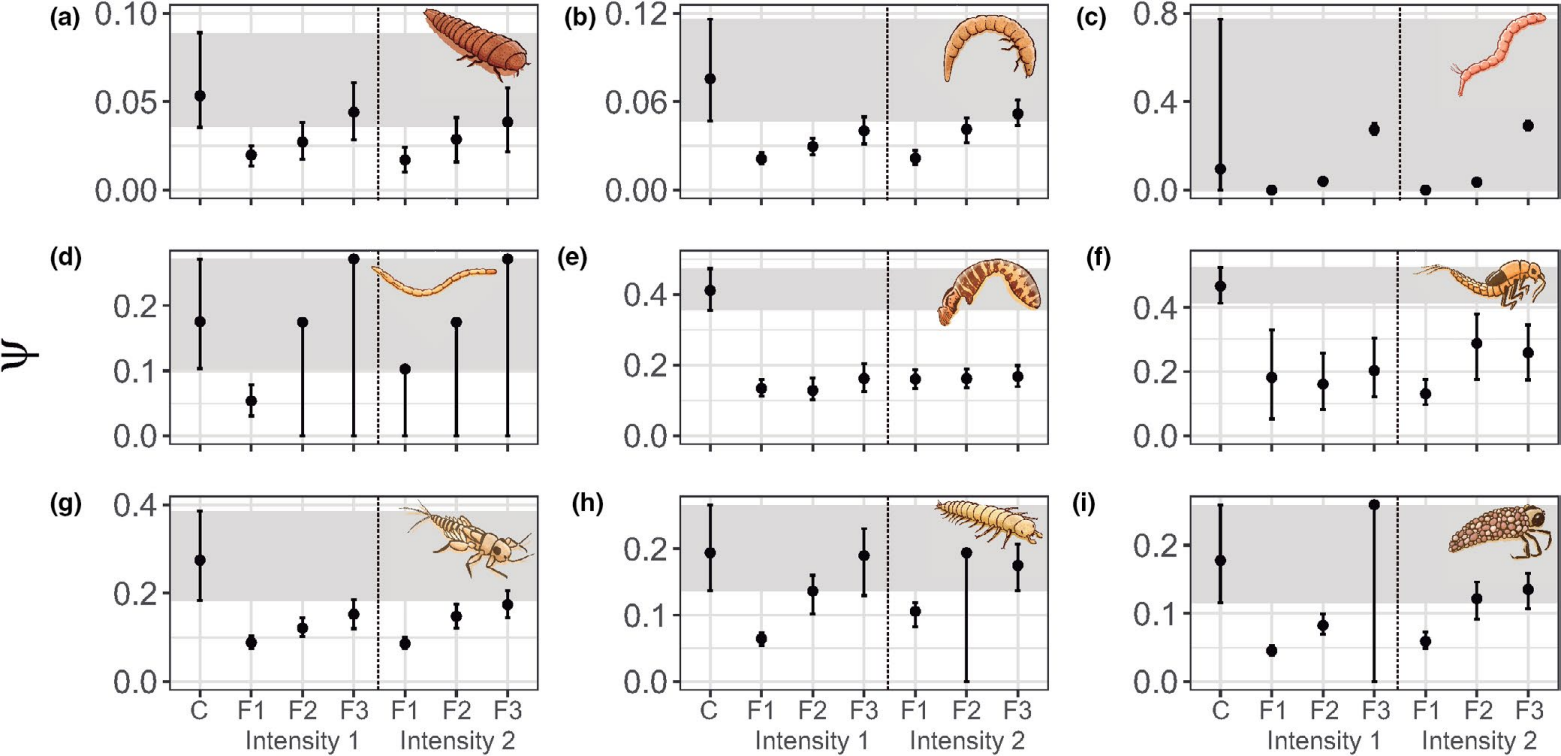
Effects of experimental treatments (disturbance intensity and frequency) on the probability of permanence, with the mean values and credibility intervals (95%). a = Depressed‐bodied Coleoptera; b = Cylindrical Coleoptera; c = Chironomidae; d = Ceratopogonidae; e = Empididae and Simuliidae; f = Baetidae; g = Depressed‐bodied insects; h = Larvae with anal claws; and i = Trichoptera shelter builders. C = control; F1, F2, and F3 = frequency of disturbance (interval in days 6, 3, and random). The gray band corresponds to the credibility interval of the control group

In general, disturbance intensity affected the permanence probabilities of the functional groups similarly (Table 3). However, the frequency of disturbance events affected the permanence probability of all groups except Chironomidae larvae (group 3). The lower frequency of disturbance events (F1: disturbance every 6 days) affected a greater number of groups when compared to the higher frequency (F2: disturbance every 3 days) or to the random frequency treatments (F3). The lower frequency reduced the permanence probabilities more strongly (Figure 4).

**TABLE 3:** Proportion of functional groups of aquatic insects affected by each experimental treatment

	Intensity 1	Intensity 2
Frequency 1	8 (88.9%)	7 (77.8%)
Frequency 2	5 (55.6%)	3 (33.3%)
Frequency 3	2 (22.2%)	2 (22.2%)

The probability of permanence for Diptera was little affected by the disturbances. Group 3 (Chironomidae) was not affected by the treatments (Figure 4c) and group 4 (Ceratopogonidae) was affected only by treatment 1 (low intensity and lower frequency, Figure 4d). Depressed‐bodied Coleoptera were less affected than those with cylindrical bodies (groups 1 and 2, respectively), for example, Baetidae nymphs (group 6, Figure 4f) were more affected than flat‐bodied insects (group 7, Figure 4g). In addition, larvae with anal claws (group 8, Figure 4h) were less affected by disturbance than Trichopterans building portable shelters (group 9, Figure 4i). Unexpectedly, the permanence probability of group 5, represented by the families Empididae and Simuliidae (Diptera), was affected by all treatments in the experiment (Figure 4e).

## 4 DISCUSSION

The increase in hydrological disturbances is expected to impact aquatic biodiversity worldwide in the coming years (Arias et al., 2021; Fischer & Knutti, 2015). Previous studies have shown that high water flow intensity and frequency can drive rapid declines in species richness and abundance of aquatic biodiversity (Brasil et al., 2017, 2018; de Resende et al., 2021; Melo et al., 2003; Resh et al., 1988). However, functional responses of tropical biodiversity, particularly regarding aquatic insects, remain poorly understood. Here, we showed different responses of aquatic insects to changes in the water flow frequency and intensity in streams of the Cerrado biome, a biodiversity hotspot expected to experience strong hydrological changes related to land use and climate change (Bowman, 2016). In general, we observed that a greater water flow intensity tends to affect the resistance (in our study represented by the permanence parameter) of almost all insect groups; however, this effect is softened over time. On the other hand, less frequent disturbances, regardless of intensity, tend to reduce the permanence of most aquatic insects over time.

Frequency affected the permanence in an opposite direction than we had hypothesized (second hypothesis) based on classical studies on disturbance in aquatic systems (Connell, 1978; Resh et al., 1988; Townsend, Scarsbrook, et al., 1997; Ward, 1989). Most of the groups that showed a reduced permanence when under less frequent disturbances (depressed bodied beetles, cylindrical beetles, Ceratopogonidae, depressed body insects, larvae with anal claws, and shelter‐building Trichoptera, Figure 4a, b, d, g, h, and i) had, in general, a greater baseline probability of recolonization than of permanence. The combination of these results shows that the effect of disturbance frequency does not imprint a general pattern in all groups, but rather depends on the functional characteristics of the organisms.

Disturbance may be an external element that reduces the effects of interactions between the organisms inhabiting the artificial substrates (Menge & Sutherland, 1987). With a higher frequency of disturbances, habitats became more susceptible to colonization because frequent disturbances had removed organisms from the artificial substrates (Burton et al., 2020), loosening competition. In this scenario, groups with selected characteristics that favor displacement and recolonization in detriment of mechanisms of resistance to disturbances may quickly colonize vacant habitats, or even increase their population sizes in a short period of time (e.g., Chironomidae), thus increasing the likelihood of permanence. On the other hand, less frequent disturbances can favor populations with high growth rates, increasing the effect of intraspecific competition (Huston, 1979; Magurran & Huston, 1995). This may explain, for example, the moderate probabilities of recolonization and permanence recorded for Empididae and Simulidae, groups with strategies to remain fixed on the stream substrate and fast life cycle (Carvalho & Uieda, 2004; Landeiro et al., 2010). Such reasoning is congruent with part of the assumptions of the intermediate disturbance hypothesis, in which the frequency of disturbances is directly related to the relaxation of interactions among species (Wilkinson, 1999).

Our study adds evidence that a generalization like “water flow intensity affected the permanence of…” is valid mainly for the period immediately after the disturbance because the effects of disturbance lose its intensity over time on macroinvertebrates. This pattern may be related to the resilience (recolonization parameter) capacity of macroinvertebrates since many recolonize substrates subject to disturbances in few hours or days (Boulton et al., 1992; Brooks & Boulton, 1991; Godoy, Queiroz, et al., 2016; Landeiro et al., 2010; Townsend & Hildrew, 1976). In addition, Poff and Zimmerman (2010) reviewed the response of aquatic organisms to changes in water flow and showed that the abundance and diversity of macroinvertebrates have mixed responses to changes in intensity of the flow regime. Although the study did not assess the abundance and diversity of aquatic insects, our results did indicate that the occurrence of insects in relation to disturbance intensity depends on time elapsed (immediately or days after the disturbance).

### 4.1 The power of the Bayesian model to detect the response of different functional groups

The individual hypothesis for recolonization and permanence of functional groups using the space‐state Bayesian model yielded at least three different response patterns: (i) the estimated parameters of permanence and recolonization support the hypothesis raised; (ii) at least one of the parameters support our hypothesis; or (iii) no parameter fit the hypothesis.

The Chironomidae and Ceratopogonidae larvae are clear examples of the first case, once their probabilities of permanence and recolonization were very close to the hypothesized probabilities. During the experiment, we found a low probability of permanence but a high and moderate probability of recolonization for Chironomidae and Ceratopogonidae, respectively. This indicates that these organisms invest primarily in a rapid recolonization of habitats, which is in line with the life cycle of these organisms. The representatives of these families usually have high densities and a relatively short life cycle (Trivinho‐Strixino & Strixino, 1999). Ceratopogonidae and Chironomidae females can have a life span of up to 70 and 22 days, respectively (Braverman, 1994), during which the females have two to four reproductive cycles (Corbi & Trivinho‐Strixino, 2006).

Only the parameters estimated for the group composed of Coleoptera larvae with depressed bodies (i.e., *Macrelmis*, *Phanocerus*, *Xenelmis*) did not support our hypotheses. Contrary to our expectation, these larvae had a low permanence during the study period accompanied by a moderate habitat recolonization capacity. An explanation for this pattern may be the mobility capacity of these larvae, and their active search for habitats (Carvalho & Uieda, 2006) that allows an alternated occurrence of these organisms in the samples.

Our hypotheses were supported by at least one of the estimated parameters of the remaining six groups. Many explanations may account for these results, and we need to highlight that some parameters may have been overestimated in some hypotheses because our previous information about the biology of some groups was limited. Despite this Hutchinsonian shortfall, the mobility of Coleoptera larvae, other uses of shelters (e.g., Trichoptera using the shelter to protect against possible natural enemies, Morse, 2009), rapid recolonization of empty habitats (Ephemeroptera and Plecoptera, Carvalho & Uieda, 2004; Landeiro et al., 2010), and the use of drift strategies for recolonization (Megaloptera and some Coleoptera and Trichoptera larvae, Godoy, Queiroz, et al., 2016; Krueger & Cook, 1984; Townsend & Hildrew, 1994) may explain the divergence between the hypothesis and patterns observed in this study.

A relevant point in our study was the way we categorized the functional groups. The groups were assembled according to life strategies based on functional traits. In addition, we established testable numerical hypotheses for predetermined parameters. Both biology of organisms and ecological theory were included in a predictive way (Verberk et al., 2013). The elaboration of these hypotheses and our experimental space‐state design made it possible for us to measure the permanence and recolonization responses separately. The separate estimation of the parameters that represent these functional responses was essential to understand how changes in flow conditions can impact the occurrence of organisms. We also directly tested the axes of resistance and resilience that summarize the fundamental theory of functional traits of aquatic insects subject to environmental disturbances (Lopez et al., 2016). Separately, functional groups respond differently to changes in the flow regimes, even groups with similar baseline permanence and recolonization values.

Understanding the response of functional groups to different disturbances is important to design theories and hypotheses that explain temporal dynamics of aquatic communities, and to describe the possible alternative states presented by communities depending on environmental stress. Despite the separate estimates of permanence and recolonization probability, the experimental design does not allow a complete separation of these two parameters and, thus, the estimated parameters may have a degree of covariance. The long‐term permanence probability may be the result of a continuous occupation of organisms, partially affected by the recolonization processes (Boulton et al., 1992; Walters, 2011). Despite this limitation, the use of a space‐state analysis reduces this covariance to acceptable limits. To control and quantify this covariance, a similar study limiting habitat recolonization is necessary. It is also noteworthy that our experimental design was based on a categorical quantification of the disturbances, leaving out a possible continuum for flow disturbance intensity and frequency. However, this qualification is common in studies that assess the effect of the disturbance on biodiversity (Poff & Zimmerman, 2010) and the levels we choose are relevant to the type of environment in which we tested our hypotheses.

### 4.2 Implication of our findings for aquatic biodiversity conservation in the Cerrado Hotspot

The evidence from our study that higher hydrological disturbance frequency can lead to reduced permanence of some insect groups (particularly EPT taxa) has some practical implications to biodiversity conservation and restoration practices in the Cerrado Hotspot. Cerrado streams usually undergo seasonal flooding (Bispo & Oliveira, 2007; Bispo et al., 2004) and land use changes near streams is accelerating in the Cerrado region (Bowman, 2016). The loss of riparian vegetation makes flooding events more frequent, especially in the rainy season. The Cerrado is also targeted for construction of small‐ to medium‐sized dams, which directly affects the hydrological dynamics of lotic systems. Moreover, climate change is predicted to condense the rainy season into shorter periods of time at a regional scale, but with more intense precipitation events in the Cerrado region (Arias et al., 2021). Considering these issues, we can expect, on a broader temporal and spatial scale, that aquatic biodiversity will experience more “hydrological disturbances” in the coming years, which will directly affect the permanence of aquatic insects in headwater streams, especially those with less vegetation cover.

Under this perspective, general strategies to conserve and restore biodiversity in the Cerrado, particularly those related to minimizing hydrological extreme events, would also benefit aquatic insects. Strategies vary from more ethical and moral arguments to more economic perspectives, for example: improving restoration and protection of key Cerrado terrestrial and aquatic habitats (including streams) while enabling agricultural expansion by using clear criteria in land use planning, increasing pasture productivity, promoting economic mechanisms of bioeconomy, and expanding the Soy Moratorium to the Cerrado for sugarcane and beef (Strassburg et al., 2017). Moreover, the territorial planning and implementation of policies for biodiversity conservation and restoration based on water, food, energy, and ecosystems (WFEE) nexus approach is particularly important for reducing hydrological disturbance in the Cerrado because the hydrology of the region can be seriously affected depending on decisions involving these dimensions (Colman et al., 2022). Beyond general strategies to conserve and restore biodiversity at large scale in the Cerrado, which can also be beneficial for aquatic insects, maintaining the riparian forest cover seems to be key in maintaining the biodiversity of aquatic entomofauna in tropical streams (Dala‐Corte et al., 2020). Particularly for some EPT groups, including the sensitive groups for hydrological disturbances that we detected here (e.g., larvae with anal claws and shelter‐building Trichoptera), as it prevents water runoff from reaching streams quickly, attenuating flow disturbances and losses of functional groups is important for these aquatic insect communities.

Climate change is predicted to condense the rainy season into shorter periods of time at a regional scale, but with more intense precipitation events in the tropics (Arias et al., 2021; Fischer & Knutti, 2015). Thus, on a broader temporal and spatial scale, we would have more intense and frequent rains, which will directly affect the permanence of aquatic insects in headwater streams, especially those with less vegetation cover. Therefore, maintaining the riparian forest cover becomes key in maintaining the biodiversity of aquatic entomofauna (Dala‐Corte et al., 2020), as it prevents water runoff from reaching streams quickly, attenuating flow disturbances and losses of functional groups important for these aquatic insect communities.

## CONFLICT OF INTEREST

The authors declare that they have no conflict of interest.

## AUTHOR CONTRIBUTIONS

**Bruno S. Godoy:** Conceptualization (lead); Data curation (lead); Formal analysis (lead); Methodology (lead); Writing – original draft (lead); Writing – review & editing (lead). **Francisco Valente Neto:** Visualization (equal); Writing – original draft (equal); Writing – review & editing (equal). **Luciano L. Queiroz:** Investigation (equal); Methodology (equal); Visualization (equal); Writing – original draft (equal); Writing – review & editing (equal). **Luis F. R. Holanda:** Conceptualization (equal); Data curation (equal); Funding acquisition (equal); Investigation (equal); Methodology (equal); Project administration (equal); Resources (equal). **Fabio O. Roque:** Visualization (equal); Writing – original draft (equal); Writing – review & editing (equal). **Sara Lodi:** Visualization (equal); Writing – original draft (equal); Writing – review & editing (equal). **Leandro G. Oliveira:** Funding acquisition (lead); Project administration (equal); Supervision (lead); Writing – original draft (equal); Writing – review & editing (equal).

### OPEN RESEARCH BADGES

This article has earned an Open Data Badge for making publicly available the digitally‐shareable data necessary to reproduce the reported results. The data is available at https://datadryad.org/stash/share/zqpXeL‐hTN64ibUkjiBzTxzPHEHftQdzLUIq55h70c.

## Supporting information

Appendix S1

## Data Availability

Data available at Dryad, https://doi.org/10.5061/dryad.ngf1vhhwb
