# Supplementary material for: Structuring functional groups of aquatic insects along the resistance/resilience axis when facing water flow changes
Source: Ecol Evol. 2022 Mar 26;12(3):e8749. doi: 10.1002/ece3.8749 (PMC8956860; doi:10.1002/ece3.8749)
Supplement: Supplementary file 1 — Appendix S1 [file ECE3-12-e8749-s001.zip › ece38749-sup-0010-AppendixS1.docx]

**Appendices**

Figures

Figure A1. Credibility interval for 95% of the posterior distribution of permanence and colonization parameters for functional group 1 – Coleoptera larvae with depressed body.

Figure A2. Credibility interval for 95% of the posterior distribution of permanence and colonization parameters for functional group 2 – Coleoptera larvae with cylindrical body.

Figure A3. Credibility interval for 95% of the posterior distribution of permanence and colonization parameters for functional group 3 – Chironomidae larvae.

Figure A4. Credibility interval for 95% of the posterior distribution of permanence and colonization parameters for functional group 4 – Ceratopogonidae larvae.

Figure A5. Credibility interval for 95% of the posterior distribution of permanence and colonization parameters for functional group 5 – Empididae and Simuliidae larvae.

Figure A6. Credibility interval for 95% of the posterior distribution of permanence and colonization parameters for functional group 6 – Baetidae larvae.

Figure A7. Credibility interval for 95% of the posterior distribution of permanence and colonization parameters for functional group 7 – Leptohyphidae, Leptophlebiidae, and Plecoptera.

Figure A8. Credibility interval for 95% of the posterior distribution of permanence and colonization parameters for functional group 8 – Gyrinidae (larvae), Megaloptera, and some Trichoptera.

Figure A9. Credibility interval for 95% of the posterior distribution of permanence and colonization parameters for functional group 9 – Calamoceratidae, Hydroptilidae, Leptoceridae, and Odontoceridae.

Models

Immediate effects

The model designed to estimate the effect of disturbance in the occurrence parameter of the genera in the R software.

#Data

library(BRugs)

occurrence<-data1 #The data1 is a table with 3 columns and *n* rows, where *n* is the number of genera. The column 1 is the occurrence of the genera in all streams before the disturbance, the column 2 is the occurrence after the disturbance, and the column 3 is the group based on functional traits of the genera.

n<-dim(occurrence)[1]

s<-data2 #Number of genera

g<-data3 #Number of functional groups

imedata<-list(occurrence=occurrence, n=n, s=s, g=g)

bugsData(imedata, fileName=file.path(tempdir(), “imedata.txt”))

#Parameters

parameters_ime<-c(‘a’, ‘b’, ‘e’, ‘effect’)

iteration<-1000

burning<-1000

thin<-5

chain<-5

#Initials

initials_ime<-function(){list(a=c(rep(sample(0:1,1)/100),g)), b=c(rep(sample(0:1,1)/100),g)), e=c(rep(sample(1:100,1),n)))}

bugsInits(initials_ime, numChains=1, fileName=“initiais_ime.txt”, digits=3, format=“E”)

#Model

sink(‘mod_ime.txt’)

cat(‘

Model{

for(j in 1:g){

a[g] ~ dbeta(0.001,0.001)

b[g] ~ dbeta(0.001,0.001)

}

for(i in 1:n){

a[occurrence[i,3]] = b[occurrence[i,3]]/e[i]

occurrence[i,1] ~ dbinom(b[occurrence[i,3]],s)

occurrence[i,2] ~ dbinom(a[occurrence[i,3]],s)

e[i] ~ dgamma(0.001,0.001)

effect[i] = exp(e[i])

}

}

’, fill=TRUE)

sink()

fit.ime<-BRugsFit(data=“imedata.txt”, inits=rep(“initiais_ime.txt”, chain), para=parameters_ime, modelFile= ‘mod_ime.txt’, numChains=chain, nIter=iteration, nBurin=burning, nThin=thin, coda=T)

Basal probability of occurrence and colonization

The model designed to estimate the basal probability of occurrence and colonization for genera in the R software. The data used are the control samples in the experiment.

#Data

library(BRugs)

hist.occurence<-data4 # The data4 is a table with *n***s* columns and *t* rows, where *n* is the number of genera, *t* is the number of episodes of sample in the experiment, and *s* is the number of streams. Each column represents the history of occurrence of individual genera in each stream, where 0 is the absence and 1 is the presence.

c<-dim(hist.occurrence)[2]

r<-dim(hist.occurrence)[1]

n<-data5 # Number of genera

f.g<-data6 # The vector representing the functional group of each genera

g<-max(f.g)

genera<-rep(1:n, each=*s*) # *s* the number of streams.

histdata<-list(hist.occurrence=hist.occurrence, c=c, r=r, n=n, f.g=f.g, g=g, genera=genera)

bugsData(histdata, fileName=file.path(tempdir(), “histdata.txt”))

#Parameters

parameters_hist<-c(‘alpha1’, ‘alpha2’, ‘beta1’, ‘beta2’, ‘theta’, ‘psi’, ‘p’)

iteration<-1000

burning<-1000

thin<-5

chain<-5

#Initials

initials_hist<-function(){list(alpha1=c(rep(sample(0:100,1),g)), alpha2=c(rep(sample(0:100,1),g)), beta1=c(rep(sample(0:100,1),g)), beta2=c(rep(sample(0:100,1),g)), theta=c(rep(sample(0:1,1)/100),n)), psi=c(rep(sample(0:1,1)/100),n)))}

bugsInits(initials_hist, numChains=1, fileName=“initiais_hist.txt”, digits=3, format=“E”)

#Model

sink(‘mod_his.txt’)

cat(‘

Model{

for(i in 1:g){

alpha1[i] ~ dgamma(0.001,0.001)

beta1[i] ~ dgamma(0.001,0.001)

alpha2[i] ~ dgamma(0.001,0.001)

beta2[i] ~ dgamma(0.001,0.001)

}

for(i in 1:n){

theta[i] ~ dbeta(alfa1[f.g[i]], beta1[f.g[i]])

psi[i] ~ dbeta(alfa2[f.g[i]], beta2[f.g[i]])

}

for(i in 1:c){

for(j in 2:r){

hist.occurence[r,c] ~ dbern(p[r,c])

p[r,c] = theta[genera[i]] + hist.occurrence[r-1,c]*psi[genera[i]]

}

}

}

’, fill=TRUE)

sink()

fit.hist<-BRugsFit(data=“histdata.txt”, inits=rep(“initiais_hist.txt”, chain), para=parameters_hist, modelFile= ‘mod_his.txt’, numChains=chain, nIter=iteration, nBurin=burning, nThin=thin, coda=T)

Effects of the intensity and frequency of disturbance in the permanence of the genera

The model designed to estimate the effects of the intensity and frequency of disturbance in the probability of permanence for genera in the R software. The data used are the experimental samples.

#Data

library(BRugs)

exp.occurence<-data7 # The data7 is a table with *n***s***f* columns and *t* rows, where *n* is the number of genera, *t* is the number of episodes of sample in the experiment, *s* is the number of streams, and *f* is the number of distinct effects in a factorial experiment. Each column represents the history of occurrence of individual genera in each stream and under specific effect of the experiment, where 0 is the absence and 1 is the presence.

c<-dim(hist.occurrence)[2]

r<-dim(hist.occurrence)[1]

n<-data5 # Number of genera

f.g<-data6 # Vector representing the functional group of each genera

exp<-data8 # Vector representing the distinctly effects in the experiment for each streams

g<-max(f.g)

f<-max(exp)

genera<-rep(1:n, each=*s**f) # *s* the number of streams.

expdata<-list(exp.occurrence=exp.occurrence, c=c, r=r, n=n, f.g=f.g, g=g, genera=genera, exp=exp, f=f)

bugsData(expdata, fileName=file.path(tempdir(), “expdata.txt”))

#Parameters

parameters_exp<-c(‘alpha1’, ‘alpha2’, ‘beta1’, ‘beta2’, ‘theta’, ‘delta’, ‘psi’, ‘p’)

iteration<-1000

burning<-1000

thin<-5

chain<-5

#Initials

initials_exp<-function(){list(alpha1=c(rep(sample(0:100,1),g)), alpha2=c(rep(sample(0:100,1),g)), beta1=c(rep(sample(0:100,1),g)), beta2=c(rep(sample(0:100,1),g)), theta=c(rep(sample(0:1,1)/100),n)), psi=c(rep(sample(0:1,1)/100),n)), delta=c(rep(sample(-50:50,1),f)))}

bugsInits(initials_exp, numChains=1, fileName=“initiais_exp.txt”, digits=3, format=“E”)

#Model

sink(‘mod_his.txt’)

cat(‘

Model{

for(i in 1:g){

alpha1[i] ~ dgamma(0.001,0.001)

beta1[i] ~ dgamma(0.001,0.001)

alpha2[i] ~ dgamma(0.001,0.001)

beta2[i] ~ dgamma(0.001,0.001)

}

for(i in 1:n){

theta[i] ~ dbeta(alfa1[f.g[i]], beta1[f.g[i]])

psi[i] ~ dbeta(alfa2[f.g[i]], beta2[f.g[i]])

}

for(i in 1:f){

delta[i] ~ dnorm(0,0.001)

}

for(i in 1:c){

for(j in 2:r){

hist.occurence[r,c] ~ dbern(p[r,c])

p[r,c] = theta[genera[i]] + hist.occurrence[r-1,c]*psi[genera[i]]*delta[exp[i]]

}

}

}

’, fill=TRUE)

sink()

fit.exp<-BRugsFit(data=“expdata.txt”, inits=rep(“initiais_exp.txt”, chain), para=parameters_exp, modelFile= ‘mod_exp.txt’, numChains=chain, nIter=iteration, nBurin=burning, nThin=thin, coda=T)
